# Pharmacological stimulation of murine and human hematopoetic stem cells

**DOI:** 10.1186/2050-6511-13-S1-A67

**Published:** 2012-09-17

**Authors:** Christian Bergmayr, Christian Balasz, Zarzar Kazemi, Filza Hussain, Thomas Bauer, Herbert Strobl, Peter Selzer, Wolfgang Strohmaier, Michael Freissmuth, Eva Zebedin-Brandl

**Affiliations:** 1 Institute of Pharmacology, Center for Physiology and Pharmacology, Medical University of Vienna, 1090 Vienna, Austria; 2 Institute of Immunology, Medical University of Vienna, 1090 Vienna, Austria; 3 Department of Radiation Oncology, Medical University of Vienna, 1090 Vienna, Austria; 4 SciPharm SàRL, 6131 Junglinster, Luxembourg

## Background

Hematopoietic stem cell (HSC) transplantation is a standard procedure in the treatment of hematological disorders and also applicable to support aggressive chemotherapy in cancer. In practice, the clinical outcome is often limited by inefficient bone marrow (BM) engraftment or by low numbers of available stem cells. It would therefore be desirable to enhance engraftment by pharmacological stimulation. HSC require several signals for successful migration into the bone marrow. One of these signals is provided by stimulation of Gα_s_[1]. Pretreatment with prostaglandin (PG) E_2_ enhances engraftment via activation of Gα_s_-coupled EP_2_ and EP_4_ receptors [2]. Treprostinil is a stable analogue of prostacyclin/PGI_2_, which acts via IP, EP_2_ and EP_4_ receptors and is approved for treatment of pulmonary hypertension. Here we tested the hypothesis that treprostinil stimulates stem cell engraftment.

## Methods

Generation of murine bone marrow-derived HSCs: Undifferentiated HSC (lineage-negative, Lin− cells) were isolated from murine BM, separated by MACS (magnetic-assisted cell sorting) and characterized by fluorescence-activated cell sorting (FACS). BM transplantation: Lin− cells were pretreated *in vitro* in the absence and presence of 10 µM treprostinil alone or in combination with 30 µM FSK for 1 h at 37°C. The cellular threshold for successful transplantation was determined by titrating the number of HSCs required to allow for survival of lethally irradiated recipient mice. Engraftment and transplantation efficiency was determined by the analysis of white blood cell counts.

## Results

Trepostinil triggered a concentration-dependent accumulation of cAMP in murine Lin− cells with an estimated EC_50_ in the range of 0.3 µM. A treprostini-induced cAMP accumulation was also observed in human HSCs, which were also shown to express the mRNA encoding IP, EP_2_ and EP_4_ receptors. Treprostinil enhanced engraftment of HSCs; this conclusion is based on the following observations: (i) mice injected with treprostinil-pretreated Lin− cells had significantly higher levels of circulating white blood cells as compared to those receiving vehicle-treated Lin− cells (p < 0.05, unpaired t-test); (ii) when pretreated and untreated Lin− cells were mixed to compete for BM reconstitution, the pretreatment with treprostinil increased transplantation efficiency 1.5–3-fold; (iii) *in vitro* pretreatment of HSCs with trepostinil reduced the minimum number of cells required to rescue a mouse; (iv) engraftment of HSCs was further enhanced when treprostinil was also administered after injection of the pretreated HSCs.

## Conclusions

Treprostinil is suitable for improving haematopoetic stem cell transplantaion. The observations allow for designing a protocol to test the compound in a clinical trial.
